# Characterization by Nano-Infrared Spectroscopy of Individual Aggregated Species of Amyloid Proteins

**DOI:** 10.3390/molecules25122899

**Published:** 2020-06-24

**Authors:** Jehan Waeytens, Vincent Van Hemelryck, Ariane Deniset-Besseau, Jean-Marie Ruysschaert, Alexandre Dazzi, Vincent Raussens

**Affiliations:** 1 Structure et Fonction des Membranes Biologiques, Université libre de Bruxelles, B-1050 Bruxelles, Belgique; Jehan.Waeytens@ulb.be (J.W.); jmruyss@ulb.ac.be (J.-M.R.); 2 Laboratoire de Chimie Physique d’Orsay, CNRS UMR8000, Université Paris-Sud, Université Paris-Saclay, F-91400 Orsay, France; ariane.deniset@gmail.com (A.D.-B.); alexandre.dazzi@u-psud.fr (A.D.); 3 Spectralys Biotech, Université libre de Bruxelles, B-1050 Bruxelles, Belgique; vincent.van.hemelryck@ulb.ac.be

**Keywords:** amyloid fibrils, aggregated species, oligomers, photothermal infrared nanospectroscopy, AFM-IR

## Abstract

Amyloid fibrils are composed of aggregated peptides or proteins in a fibrillar structure with a higher β-sheet content than in their native structure. To characterize them, we used an innovative tool that coupled infrared spectroscopy with atomic force microscopy (AFM-IR). With this method, we show that we can detect different individual aggregated species from oligomers to fibrils and study their morphologies by AFM and their secondary structures based on their IR spectra. AFM-IR overcomes the weak spatial resolution of usual infrared spectroscopy and achieves a resolution of ten nanometers, the size of isolated fibrils. We characterized oligomers, amyloid fibrils of Aβ42 and fibrils of α-synuclein. To our surprise, we figured out that the nature of some surfaces (ZnSe) used to study the samples induces destructuring of amyloid samples, leading to amorphous aggregates. We strongly suggest taking this into consideration in future experiments with amyloid fibrils. More importantly, we demonstrate the advantages of AFM-IR, with a high spatial resolution (≤ 10 nm) allowing spectrum recording on individual aggregated supramolecular entities selected thanks to the AFM images or on thin layers of proteins.

## 1. Introduction

Alzheimer’s disease (AD) is the most prevalent form of neurodegenerative dementia and affects more than 50 million patients worldwide. This number will double every 20 years to reach > 150 million of cases around 2050 [1]. AD is characterized by brain cell destruction, memory loss, and deterioration of cognitive and behavioral processes. One of the characteristic histopathological markers of AD is the presence of proteinaceous deposits in the brains of patients, amyloid plaques localized outside neurons, and synaptic connections [2,3] and intraneuronal neurofibrillary tangles containing hyperphosphorylated Tau protein [4]. The plaques of amyloid fibrils are mainly composed by a 39-to-43-residue misfolded peptide, the amyloid β peptide (Aβ). This peptide is a cleavage product of the amyloid precursor protein (APP) a membrane protein [5,6]. Aβ40 and Aβ42 are the main components of amyloid plaques [7].

Parkinson’s disease (PD) is the second most frequent neurodegenerative disease in humans with symptoms of aging-related movement disorder. PD is characterized by the aggregation of α-synuclein (α-syn) into fibrillar assemblies in nerve cells [8]. α-syn, a 140-residue presynaptic protein, is a natively unfolded structure but aggregates into highly ordered amyloid fibrils, amorphous aggregates or various oligomers [9].

However, experiments aiming at establishing a direct causal relationship between amyloid protein deposition (fibrils) and neurodegeneration have failed [10]. Recently, small soluble oligomeric or protofibrillar assemblies of amyloid proteins have shown to be the most toxic to neurons and their vital interconnections [11]. Nevertheless, insoluble fibrillar aggregates can induce an inflammatory response, a process that actively contributes to the toxicity of amyloidogenic proteins [12,13,14]. It seems that most amyloid peptides and proteins can adopt several transitory conformations, and the study of this polymorphism has become crucial. This remains a challenge because of the great difficulty to stabilize and purify these transitory species without structural modification.

Two methods have gained popularity in the study of protein aggregation. On the one hand, infrared spectroscopy, specifically in attenuated total reflection (ATR), can characterize the secondary structure of aggregated proteins and therefore follow conformational changes during aggregation kinetics. On the other hand, atomic force microscopy (AFM) provides the morphology, distribution, and some mechanical properties [15] of species formed during the aggregation. Recently, an innovative tool called AFM-IR, a photothermal technique based on the coupling of AFM and infrared thanks to quantum cascade lasers (QCL), allows collecting images and infrared spectra at the nanometer scale [16]. This tool is very promising for the characterization of amyloid fibrils and other protein aggregation stages, as it can correlate all the information contained in AFM images with the secondary structure and other information given by the infrared spectra. Some results using AFM-IR on different amyloid fibrils have been published [17,18,19,20,21], but only a couple have looked at different aggregated species [17,18]. Our goal is to study the differences in conformations between protein aggregates (oligomers, fibrils, and amorphous aggregates) by AFM-IR and to confirm that spectra obtained by this recent method are comparable with those collected with ATR-FTIR. This should demonstrate that AFM-IR is well-adapted to study protein aggregates at the individual level. Moreover, we show that out the chemical composition of the prism surfaces used in AFM-IR for the study of amyloid fibrils is important. Some artefactual surface effects have been observed on both Aβ and α-syn fibrils. Therefore, this should be considered for future amyloid fibrils and maybe other protein studies by AFM-IR.

## 2. Results

### 2.1. AFM-IR Characterization of Oligomers and Fibrils of Aβ42

**Figure 1:**
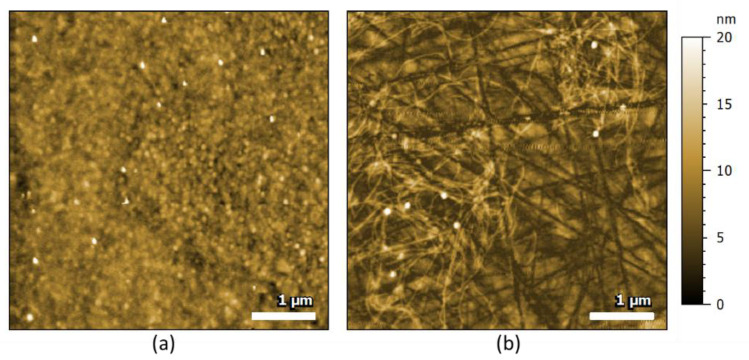
AFM topography of the Aβ42 samples at different incubation times; representative images of three images for each condition. (**a**) Just after the addition of a HCl 10 mM solution, the sample displays oligomers characterized by circular forms (white dotted shapes). (**b**) After one week of incubation at 37 °C in 10 mM HCl, we observe long fibrils with a diameter of around 10 nm as expected for amyloid fibrils (fibrils are the brighter filaments on the surface, while the scratches of the prism are the darker broad straight lines. Some oligomers remain visible as white dotted spots).

The aggregation kinetics of Aβ42 peptide are characterized by a lag phase, containing mainly monomers and oligomers, followed by a rapid increase in amyloid fibrils formation until a plateau where all or most Aβ42 is in a fibrillar form. To ensure the monomeric form of our sample as a starting point, Aβ42 was first treated with hexafluoroisopropanol (HFIP) and then dissolved in dimethylsulfoxide prior to aggregation. To evaluate the ability of AFM-IR to differentiate different species of Aβ42, we prepared oligomers at the very beginning of the aggregation kinetics and fibrils after a longer incubation (at least one week, see Materials and Methods). At the beginning of the aggregation, and probably due to the time taken to manipulate the sample, Aβ42 was not anymore entirely composed of monomers, but spherical shape oligomers were clearly present (Figure 1, left). The surface was uniformly covered by these oligomers. After 1 week of aggregation at 37 °C, we could observe amyloid fibrils with a typical diameter of 10 nm and a length greater than 1 µm. We observed, at this nanomolar concentration, isolated fibrils on the surface (Figure 1a). Some spherical entities were still visible. The long linear scratches visible on the figure are due to the cleaning process of the prism used for AFM-IR experiments; here zinc sulfide (ZnS; see Materials and Methods) is a relatively soft material.

**Figure 2:**
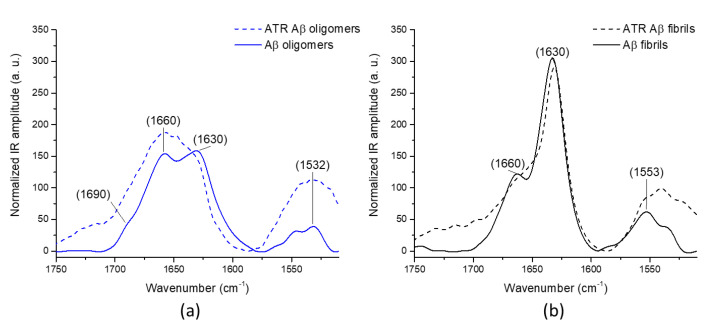
IR spectra of the oligomers and fibrils of Aβ species showed in Figure 1, normalized on the area of the amide I band. (**a**) The solid line corresponds to the averaged AFM-IR spectrum of 10 spectra recorded on different oligomers from Figure 1a and the dashed line is the spectrum obtained by ATR-FTIR (**b**) the solid line corresponds to the averaged AFM-IR spectrum of 25 different spectra recorded on different fibrils from Figure 1b, and the dashed line is the spectrum obtained by ATR-FTIR.

In Figure 2, we display the AFM-IR spectra of both species, obtained with a bottom-up illumination through a prism and the ATR-FTIR spectra. The spectrum of oligomers (Figure 2a) shows two major bands. The one located around 1660 cm^−1^ corresponds to random or α-helical structure. The band present at 1630 cm^−1^ is characteristic of β-sheet structure. We also observed a shoulder at 1690 cm^−1^ absent of the fibrils’ spectrum. The concomitant appearance of these two bands and their relative intensities indicates the presence of an anti-parallel β-sheet structure for the oligomeric state. Amyloid fibrils (Figure 2b) have a higher β-sheet content, as indicated by the higher intensity of the IR band at 1630 cm^−1^. The 1690 cm^−1^ shoulder band completely vanishes. On the amide II band, we can also observe that the maximum of the band shifts. On the oligomer, the amide II band shows a band at 1531 and a shoulder at 1547 cm^−1^. For the fibrils, the maximum is at 1552 and a shoulder appears at 1536 cm^−1^. The decrease in the 1531 cm^−1^ band with an increase in the 1552 cm^−1^ band is also a typical signature of the aggregation of protein from oligomers to fibrils [22] and confirmed the major structure for fibrils is parallel β-sheet structure. These two distinct secondary structures for oligomers and fibrils have already been observed for Aβ42 and other amyloid proteins by ATR-FTIR [23,24,25,26].

### 2.2. Comparison with ATR-FTIR and ThT Fluorescence

**Figure 3:**
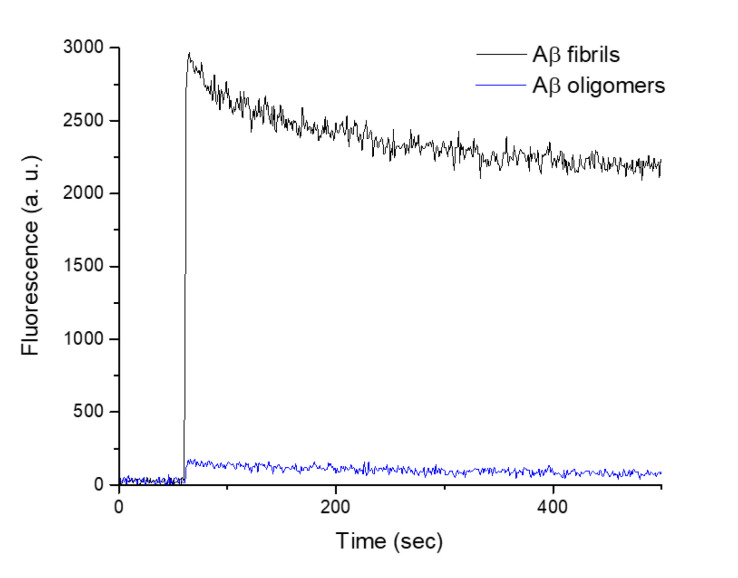
Characterization of Aβ oligomers and fibrils by ThT fluorescence. The build-up curve of the ThT fluorescence in function of time. The sample was added after 60 s for the ThT fluorescence curve.

The characterizations of oligomers and fibrils were also done using reference methods to compare and confirm our AFM-IR observations. Infrared spectra acquired by attenuated total reflection Fourier transform infrared spectroscopy corresponding to fibrils (Figure 2b, dashed line) displays a sharp band centered at 1630 cm^−1^, specific to amyloid β-sheet structure (parallel β-sheet), as previously described and in good agreement with the AFM-IR spectrum (Figure 2b solid line). In Figure 2a, corresponding to Aβ oligomeric form, the maximum of the band is around 1660 cm^−1^ corresponding to α-helix or random structures with a shoulder at 1630 cm^−1^, already indicating some β-sheet structure. For the amide II band, the bands are centered at 1540 cm^−1^ for both oligomers and fibrils, with clear shoulders at 1532 cm^−1^ for the oligomers and 1553 cm^−1^ for the fibrils; the same trend is observed in the AFM-IR spectrum. The difference in relative intensities of the two main bands between the two experiments might be due to slight differences in incubation time and by the fact that in ATR-FTIR measurements the entire sample is recorded averaging the spectral features of all species present (not only oligomers or fibrils). This demonstrates one of the biggest advantages of AFM-IR: IR measurement is done only on a single object selected in the topography by AFM. We performed a thioflavin-T (ThT) fluorescence assay on the same samples. ThT is known to fluoresce strongly in presence of β-rich amyloid structure with excitation and emission maxima at 440 and 490 nm, respectively, and free ThT exhibits only weak fluorescence [27]. In the presence of amyloid fibrils, we observed a large increase in fluorescence (Figure 3 black curve) after adding the fibrils in ThT solution (addition time 60 s). For oligomers, the build-up of fluorescence intensity is at least 10 times lower. This difference was expected, as we can see on both AFM-IR and ATR-FTIR (Figure 2a) spectra; oligomers do not have amyloid β-sheet structure but instead anti-parallel β-sheet, not recognized by ThT [28].

### 2.3. Substrate Effect of ZnSe on the Deposition of Amyloid Fibrils

**Figure 4:**
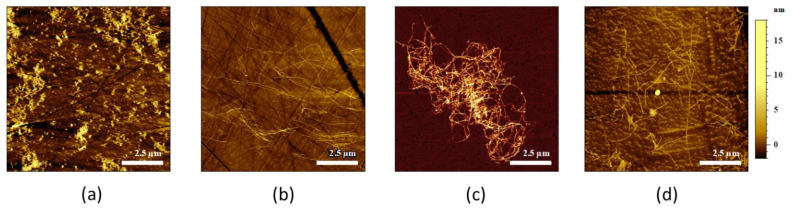
AFM images of Aβ42 on different substrates. (**a**) ZnSe prism, (**b**) ZnS, (**c**) Au and (**d**) diamond. We observed on the ZnSe prism amorphous aggregates, and on all the other surfaces well-deposited long amyloid fibrils.

Amyloid fibrils are known to be very stable species; nevertheless, in some specific cases, we observed a destabilization of the fibrils into amorphous aggregates [29,30]. However, AFM-IR measurements using bottom-up illumination configuration require the use of a specific prism (see Materials and Methods). The most commonly used matter for these prisms is zinc selenide (ZnSe). To illustrate the impact of the chemical nature of the surface, four different substrates are tested. The same sample of Aβ42 fibrils was deposited on different substrates: ZnSe prism, ZnS prism, gold (Au) substrate and synthetic diamond (Figure 4, from left to right). On the one hand, we observed on ZnS, Au, and diamond substrates well-shaped amyloid fibrils with a diameter of 10 nm and length larger than one micron. On the other hand, with ZnSe prism, we observed short fibrillar species different in shape and size. This different morphology was previously observed by other researchers and attributed to amorphous aggregates [30] or aggregates induced by metallic ions [31]. Amorphous aggregates are common to most proteins, even amyloidogenic ones when they fail to form amyloid fibrils [30]. The sample used here was the same on the four substrates and deposited at the same moment following the same protocol. The only difference is in the substrate. Gold and diamond are known to be good surfaces for biological samples, as they are mostly inert and therefore biocompatible; ZnS also seems to be well-adapted for amyloid fibril analysis as the morphology of the sample is similar on this substrate.

**Figure 5:**
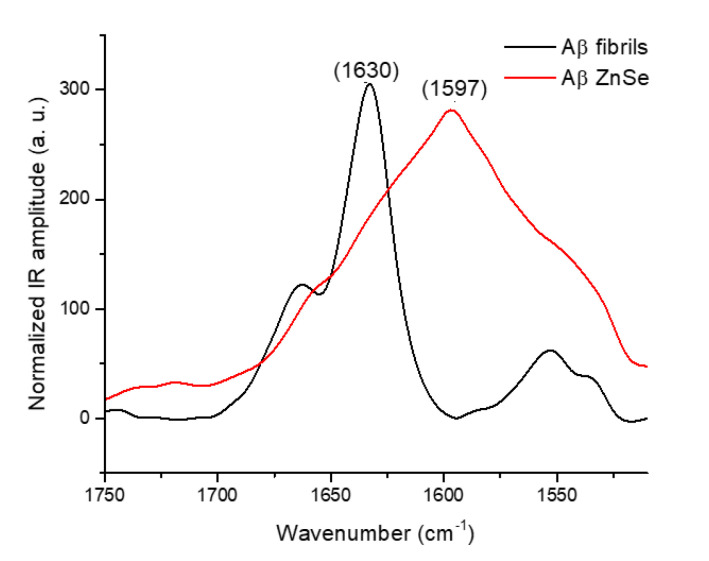
AFM-IR spectra of the Aβ different species, normalized on the amide I area for the fibrils and from 1650 and 1550 cm^−1^ for Aβ on ZnSe to obtain a comparable scale. The black curve corresponds to the fibrils on the ZnS prism. The ed curve corresponds to fibrils (from the same batch) deposited on the ZnSe prism that changed their structure to amorphous aggregate. The topography of the different species is available in Figure 4a,b respectively.

The difference does not only appear at the level of morphology but also on the secondary structure as revealed by AFM-IR. Aβ42 amyloid fibrils on ZnS prism (Figure 5, black curve) display a typical amyloid fibril spectrum with a sharp band at 1630 cm^−1^ for the parallel β-sheet, as previously discussed. Aggregates observed on the ZnSe prism show a very large band, spanning 1700 to 1500 cm^−1^. This band is difficult to explain for a proteinaceous sample; nevertheless, it demonstrates that Aβ fibril structure is not conserved on ZnSe substrate.

**Figure 6:**
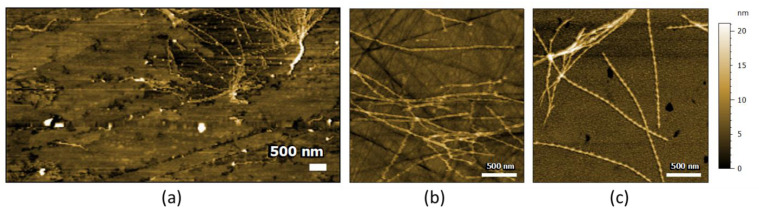
AFM images of α-synuclein fibrils on different substrates: (**a**) ZnSe prism, (**b**) ZnS prism and (**c**) Au substrate. We observed on the ZnSe prism amorphous aggregates with a few residual fibril-like structures on the top-right corner, and on all the other surfaces well-deposited long amyloid fibrils.

To confirm this effect on other amyloid fibrils, we tested different surfaces using full-length α-synuclein (α-syn), the protein responsible for Parkinson’s disease. α-syn fibrils were deposited on ZnSe prism, ZnS prism and Au substrate (Figure 6, from left to right). On ZnS and Au substrates, we obtained a typical amyloid fibril morphology with a length > 1 µm and a diameter of 10 nm. Due to the better AFM setup, we can also record a nice periodicity due to the twist in the fibrils. On the ZnSe prism, some fibrils were still present but with diameters different than 10 nm (more or less); the twist in the fibril is no longer observable, and there is a layer of protein covering the prism (Figure 6a). The ZnSe surface seems once more to induce a restructuring of α-syn fibrils.

**Figure 7:**
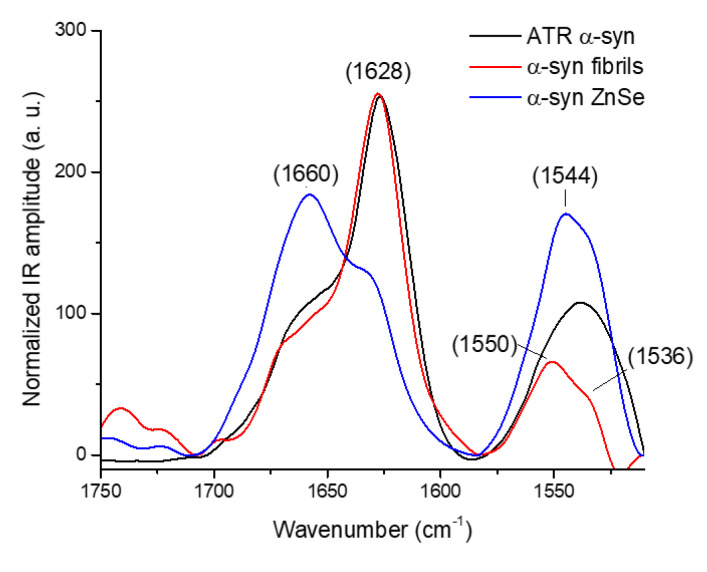
AFM-IR spectra of the α-synuclein different species, normalized on the area of the amide I band. The red curve corresponds to the fibrils on the ZnS prism. The blue curve corresponds to fibrils from the same batch deposited on the ZnSe prism. The topography of the different species is available in Figure 6a,b respectively. The black curve is the reference spectrum for fibrils obtained by ATR-FTIR.

We also acquired AFM-IR spectra for the α-syn fibrils on ZnSe and ZnS prisms. In Figure 7, the black curve represents the amyloid fibrils measured by ATR-FTIR used as a reference, and the red spectrum represents α-syn fibrils deposited on the ZnS prism. On ZnS, not only is the morphology conserved (Figure 6b), but also the IR spectrum, with a major band centered at 1628 cm^−1^ as expected for amyloids. The blue curve was obtained on a ZnSe prism. The spectrum changes drastically. Two bands are present, the major one around 1660 cm^−1^ and the second at 1630 cm^−1^. A large decrease in the β-sheet content due to a restructuring of the α-syn amyloid fibrils, similar to Aβ42 fibrils, is therefore also induced by ZnSe. On the amide II band, we can observe a decrease in the maximum wavenumber, which is at 1550 cm^−1^ for the fibrils on ZnS and at 1544 cm^−1^ for restructured fibrils on the ZnSe; both spectra have a shoulder peak at 1536 cm^−1^.

## 3. Discussion

Amyloid fibrils possess a high content of ordered cross-β structure. During the aggregation process, several species may coexist at the same time as oligomers, protofibrils and fibrils. To differentiate oligomers from fibrils, we proposed here to use a combination of two commonly used techniques for the characterization of those assemblies called AFM-IR, atomic force microscopy–infrared spectroscopy. This study was performed on oligomers and amyloid fibrils of Aβ42 and fibrils of α-syn.

For Aβ oligomers, the IR spectrum displays an amide I band centered around two wavenumbers (Figure 2a), one at 1660 cm^−1^ attributed to a random structure or α-helix and a second band at 1630 cm^−1^ with a shoulder at 1690 cm^−1^, characteristic of anti-parallel β-sheet structure. This is the expected structure for oligomers [23]. The AFM topography of such species is spherical in shape, with a diameter of around 20 nm. Comparison with ATR-FTIR spectrum (Figure 2a) demonstrates one of the advantages of AFM-IR. Indeed, in ATR-FTIR without further separation, the spectrum contains contributions of all the species present in the sample. Meanwhile, in AFM-IR, one can specifically select oligomers based on AFM image and topography.

For Aβ fibrils, the IR spectrum shows a major sharp band at 1630 cm^−1^ with a second band at 1662 cm^−1^; the structure is mainly a parallel β-sheet structure with some random or α-helix structures. The AFM topography shows long fibrils with a length of a few µm and a diameter of 10 nm. For α-syn fibrils, the AFM-IR spectrum has also a major sharp band at 1628 cm^−1^, corresponding to a high content of β-sheet, and a second band at 1660 cm^−1^ that could be attributed to either α-helix or random structures. We clearly showed that, in the case of α-syn and Aβ fibrils, the parallel β-sheet structure is confirmed in infrared spectroscopy by the presence of the 1630 cm^−1^ band and the absence of the 1690 cm^−1^ shoulder [26]. Moreover, the results of fluorescence with thioflavin-T for Aβ42 also indicate an increase in parallel cross β-sheet content for fibrils [28]. This demonstrates another big advantage of AFM-IR, its ability to follow the secondary structure of supramolecular species with a thickness around 10 nm or even below. However, to obtain those results using AFM-IR in bottom-up configuration, a ZnS prism was used, when the most widely used material is ZnSe.

Indeed, we observed an effect of the surface used to study the samples by AFM-IR. We tested four different surfaces: ZnSe, ZnS, gold and diamond. Gold and diamond surfaces are known surfaces for the study of amyloid fibrils and were used as a reference material. Thanks to the topography, we observed that, during the deposition of amyloid fibrils on the ZnSe substrate, Aβ fibrils changed their structure from well-defined long amyloid fibrils into short fibril-like amorphous aggregates. For the α-syn fibrils, the fibrils’ structures changed to a layer of protein without defined morphology; some residual fibrils can be seen, but with a diameter thinner than the initial amyloid fibrils. On the other surfaces, ZnS, Au and diamond, we observed long fibrils with a diameter of 10 nm and a length of few µm, typical of amyloid fibrils. On ZnSe, the amorphous aggregates have higher variation in length, diameter and shape.

This change is further confirmed by the local infrared spectrum of these species. The band at 1630 cm^−1^, corresponding to the β-sheet structure of the amyloid fibrils, decreases, with a new large band at 1600 cm^−1^ covering both amide I and II appearing for the Aβ42 peptide and an increase in the 1660 cm^−1^ band for α-syn (α-helix or random structure). Unfortunately, right now we cannot explain this strong effect of the ZnSe surface. It has been shown that zinc ions (Zn^2+^) can bind to Aβ and redirect its assembly from amyloid fibrils toward amorphous aggregation [32], but, because the same effect is not observed on ZnS surface, it is difficult to incriminate zinc ions here. It could be related to the ionic charge of the surface during the deposition phase in the presence of water. Ruggeri et al. developed a microfluidic deposition system to avoid restructuring of the fibrils on ZnSe [33]. Their microfluidic system spread microdroplets with a millisecond evaporation time on the ZnSe prism and seems to avoid perturbing the structure and shape of the amyloid.

In conclusion, some surfaces might induce restructuring of amyloid samples, and we show the advantages of using the AFM-IR technique for the study of protein aggregation. For complex mixtures, a selection based on the morphology can be done with the AFM topography. Based on this selection, the exact infrared signature can be obtained for different species without further need for purification or separation. Thanks to AFM-IR, intermediated species formed by amyloid proteins during their aggregation pathways will be detected and characterized. A better characterization with both morphological and structural information on the protein during aggregation can improve our knowledge of amyloses and potentially help to develop new therapeutic approaches.

## 4. Materials and Methods

### 4.1. Aβ and α-syn Sample Preparation

Synthetic Aβ42 peptides from Bachem (Bubendorf BL, Switzerland) were dissolved in cold hexafluoroisopropanol (HFIP) at a concentration of 2 mg/mL and incubated 1 h at room temperature. HFIP was removed first under nitrogen flow and then using a Speed Vac (Fisher Thermo Savant, Waltham, MA, US) for 1 h. Before use, peptides were dissolved in dimethylsulfoxide (final peptide concentration: 5 mM) and then diluted to a final concentration of 100 µM in HCl 10 mM (final measured pH ~2). Samples of oligomers were taken directly after HCl addition and analyzed directly. Peptides were incubated in solution at 37 °C during at least 1 week.

Fibrils of α-synuclein (sample courtesy of Dr Luc Bousset) were prepared as previously described [34] and were diluted 1000 times in mQ water before use in AFM-IR.

### 4.2. AFM-IR Sample Preparation and Measurements

Samples were diluted with mQ water at a final concentration between 500 and 100 nM. A quantity of 1 µL was deposited on ZnS and/or ZnSe prisms and dried at room temperature under light airflow. The samples were analyzed in enhanced contact resonance mode with a nanoIR1 from Bruker with a bottom-up illumination configuration. This is equipped with a QCL laser (Daylight Solution, San Diego, CA, USA) with one chip centered at 1650 cm^−1^ and a silicon AFM tip with a spring constant of 0.03 N/m (HQ:CSC38/Al-BS). Both ZnS and ZnSe prisms used were obtained from Bruker. The surface was cleaned with isopropanol and water before deposition. AFM-IR spectra presented are the average of 25 different spectra acquired on different fibrils (and 10 spectra from different oligomers) with all orientations, smoothed with a double-Gaussian filter with a cut-off of 2.5 cm^−1^ with Mountains Map 8 (Digital Surf, Besançon, France) and normalized on the area of the amide I band (from 1700 to 1600 cm^−1^).

### 4.3. ATR-FTIR Measure of Aβ and α-syn

Attenuated total reflection Fourier-transform infrared spectra were recorded on a Bruker (Billerica, MA, USA) Equinox 55 infrared spectrophotometer equipped with an MCT Detector at a resolution of 2 cm^−1^. The spectrometer was continuously purged with dry air. The sample was dried on a Specac (Orpington, UK) diamond with a flux of dry air, and 128 scans were averaged for the sample and background. The spectra were normalized on the area of the amide I band (from 1700 to 1600 cm^−1^).

### 4.4. ThT Fluorescence

For each experiment, 4.5 μg of Aβ peptide was added after 60 s in 1 mL of ThT solution (5 µM of ThT, 50 mM glycine pH 8.5 filtered with 0.2 µm pores) under gentle agitation. The fluorescence was measured on a PTI QuantaMaster (Horiba, Kyoto, Japan) fluorimeter, with the excitation wavelength at 450 nm and 5 nm of bandwidth and the emission wavelength at 482 nm and 10 nm of bandwidth.
